# Colistin Resistance in *Escherichia coli* and *Salmonella enterica* Strains Isolated from Swine in Brazil

**DOI:** 10.1100/2012/109795

**Published:** 2012-08-22

**Authors:** Adriano Savoia Morales, Juliana Fragoso de Araújo, Vasco Túlio de Moura Gomes, Adrienny Trindade Reis Costa, Dália dos Prazeres Rodrigues, Thais Sebastiana Porfida Ferreira, Pedro Henrique Nogueira de Lima Filsner, Maria Roberta Felizardo, Andrea Micke Moreno

**Affiliations:** ^1^Laboratório de Sanidade Suína e Virologia, Faculdade de Medicina Veterinária e Zootecnia (FMVZ), Universidade de São Paulo (USP), 05508 270 São Paulo, SP, Brazil; ^2^Instituto de Pesquisas Veterinárias Especializadas-IPEVE, Rua Esmeralda, 786, 304111-191 Belo Horizonte, MG, Brazil; ^3^Laboratório de Enterobactérias, FIOCRUZ, Avenida Brasil, 4365, Pavilhão Rocha Lima, 3°andar, Manguinhos, 21040-360 Rio de Janeiro, RJ, Brazil

## Abstract

Reports about acquired resistance to colistin in different bacteria species are increasing, including *E. coli* of animal origin, but reports of resistance in wild *S. enterica* of different serotypes from swine are not found in the literature. Results obtained with one hundred and twenty-six *E. coli* strains from diseased swine and one hundred and twenty-four *S. enterica* strains from diseased and carrier swine showed a frequency of 6.3% and 21% of colistin-resistant strains, respectively. When comparing the disk diffusion test with the agar dilution test to evaluate the strains, it was confirmed that the disk diffusion test is not recommended to evaluate colistin resistance as described previously. The colistin MIC 90 and MIC 50 values obtained to *E. coli* were 0.25 **μ**g/mL and 0.5 **μ**g/mL, the MIC 90 and MIC 50 to *S. enterica* were 1 **μ**g/mL and 8 **μ**g/mL. Considering the importance of colistin in control of nosocomial human infections with Gram-negative multiresistant bacteria, and the large use of this drug in animal production, the colistin resistance prevalence in enterobacteriaceae of animal origin must be monitored more closely.

## 1. Introduction

 Polymyxyns are a group of polypeptide antibiotics positively charged that derive from various species of *Paenibacillus (Bacillus) polymyxa. *Out of five polimixyns originally described, two have been used in the clinical setting, polimixyn B and polymyxyn E, also known as colistin [1]. 

 Colistin was discovered in 1949 and was later cautiously used clinically because of reported high incidence of nephrotoxicity and neurotoxicity. There are two forms of colistin commercially available: colistin sulfate for oral and topical use and colistimethate sodium for parenteral use; both can be delivered by inhalation, but colistin sulfate, and not colistimethate sodium, should be used for susceptibility testing [2, 3].

 Human Infections caused by multiresistant Gram-negative bacteria such as *Pseudomonas aeruginosa, Acinetobacter baumannii *and* Klebsiella pneumoniae* are increasing worldwide. In these cases, colistin has been attracting great interest because of its significant activity against these agents and low resistance rates to it [2].

 In veterinary medicine, colistin sulfate is mainly used in oral preparations, due to its excellent activity against *Escherichia coli *and* Salmonella enterica*, low frequency of resistance, and poor absorption after oral administration, especially in pigs and poultry production, although in the last few years the *E. coli* resistant to colistin is becoming more common [4]. Mechanisms of acquired colistin resistance have been described in different Gram-negative bacteria, and, more extensively, in *A. baumannii* and *Salmonella *Typhimurium [1, 5].

 The disk diffusion test that is largely used in veterinary laboratories does not seem to be a reliable method for detection of colistin resistance. A previous study [4] describes the use of a disk prediffusion method as a rapid test to determine susceptibility of pig *E. coli* isolates in Belgium, but occurrence of colistin resistance in *Salmonella enterica* strains from swine have not been described yet. In Brazil, there are no reports of colistin resistance in *E. coli* and *Salmonella enterica* of animal origin. The objective of this study is to evaluate colistin resistance in *E. coli* and *Salmonella enterica* isolated from pigs from commercial swine herds in Brazil.

## 2. Material and Methods

### 2.1. Collecting and Isolating Strains

 One hundred and twenty-six *E. coli* strains isolated from pigs presenting either postweaning diarrhea or oedema disease were selected from ten different Brazilian swine herds. One hundred and twenty-four *Salmonella enterica* subspecies enterica strains were isolated from pigs presenting enterocolitis (45/124) from nine swine herds. Carcasses, feces, and lymph nodes of healthy pigs were examined at four Brazilian slaughterhouses representing eight different swine herds (79/124). There was no correlation between the properties or animals where the strains of *E. coli* and Salmonella enterica were isolated.

 For *E. coli* isolation, feces and gut samples were inoculated on Columbia agar (Difco-BBL, Detroit, MIUSA) supplemented with 5% sheep blood and MacConkey Agar (Difco-BBL, Detroit, MI/USA), incubated for 24 h at 37°C.

*S. enterica* isolation protocol consisted in inoculation of feces, mesenteric lymph nodes, and carcass swabs into 100 mL of tetrathionate broth (Difco-BBL, Detroit, MI/USA) and incubation at 37°C for 48 h, subculturing 10 *μ*L of the tetrathionate broth into 10 mL Rappaport Vassiliadis (RV) broth (Difco-BBL, Detroit, MI/USA) and incubation at 37°C for 24 h, then inoculating xylose-lysine-tergitol-4 (XLT4) agar plates with 10 *μ*L of the RV broth, and incubation for 24 h at 37°C [6]. The isolates were identified as *E. coli* or *S. enterica* by colony morphology and standard biochemical methods [7].

### 2.2. Characterization of Strains

 Isolates identified as *E. coli* were characterized using polymerase chain reaction (PCR) as previously described [8]. For this study one hundred and twenty-six ETEC strains positive to one or more virulence genes related to postweaning diarrhea or oedema disease as F18 and F4 fimbria and heat-labile LT, heat-stable STa, and Stx2e toxins were selected (data not shown).

 Isolates identified as *S. enterica* through biochemical tests were submitted to serotyping with antigenic characterization based on the Kauffmann-White [9] at Fundação Instituto Oswaldo Cruz (Fiocruz, Rio de Janeiro). 

### 2.3. Colistin Susceptibility Tests

 Antimicrobial sensitivity testing was carried out using two different techniques: the agar dilution method [10] and the Kirby Bauer disk diffusion test (Oxoid Ltd., Cambridge/UK). Colistin sulfate powder was obtained from Sigma Chemical (St. Louis, Mo/EUA.) and all tests were performed in Mueller Hinton agar (Difco-BBL, Detroit, MI/USA). 

The minimum inhibitory concentration (MIC) was determined as the lowest concentration that inhibited visible growth. The strains were considered to have acquired resistance when their MIC was higher than the wild type cut-off value (MIC > 2*μ*g/mL) [4].

The disk diffusion test was performed with tablets of 10 *μ*g (Oxoid Ltd., Cambridge/UK) according to the CLSI guidelines [10]. Growth inhibition zone diameters were measured manually. Interpretative criteria to determine clinical resistance were based upon breakpoints described previously [11]-resistant ≤ 11 mm and susceptible ≥ 14 mm. *E. coli* ATCC 25922 and *S*. Typhimurium ATCC 14024 were used as control strains in all performed tests [12].

## 3. Results

### 3.1. *Escherichia coli* Strains

Using the agar dilution method, eight *E. coli* strains (6.3%) were considered resistant to colistin (Table 1). MIC 50 and MIC 90 values observed were 0.25 *μ*g/mL and 0.5 *μ*g/mL, respectively. When evaluating the disk diffusion test results (Table 2), four strains classified as resistant (3.2%), 37 with intermediate susceptibility (29.4%) and 85 susceptible strains (67.4%) were observed.

### 3.2. *Salmonella enterica* Strains

From 124 *S. enterica* strains, 81 were classified as serotype Typhimurium, 13 as serotype London, 11 as serotype Anatum, eight classified as *S. enterica* subspecies *enterica* (O:4,5:-:1,2), seven as serotype Choleraesuis, three as serotype Infantis and one as serotype Bredeney. The distribution of resistant strains according to serotype is presented in Table 3. Using agar dilution method 26 (21%) *S. enterica* strains were considered resistant to colistin (Table 1). Observed MIC 50 and MIC 90 values were 1 *μ*g/mL and 8 *μ*g/mL, respectively. When analyzing disk diffusion test results (Table 2), five strains classified as resistant (4%), 29 with intermediate susceptibility (23.4%), and 90 susceptible strains (72.6%) were observed.

## 4. Discussion

The results obtained in this study confirm that the disk diffusion method is not the recommended test to monitor colistin resistance, since only 50% of *E. coli* and 20% of *S. enterica* colistin resistant strains were detected using this test. Poor results using the disk diffusion method to detect colistin resistance had been previously described [4].

Using the agar dilution test, which is considered the gold standard for colistin evaluation, 6.3% of *E. coli* and 21% of *S. enterica* tested strains resistant to colistin were detected. The frequency of *E. coli* resistant strains is similar to those described by Boyen et al. [4], who report 9.6% (15/157), and have also been reported before in *E. coli* of animal origin [13, 14].

Boyen et al. [4] described that the published MIC values for human use do not predict clinical efficiency of colistin when used in animal oral formulations. Following values calculated by Burch [15], for a feed concentration of 66 ppm of colistin, the antimicrobial will reach bactericidal concentration in the porcine jejunum for strains with a MIC of 8 *μ*g/mL, but not for strains with an MIC of 16 *μ*g/mL. The MIC values observed in this study in *E. coli* resistant strains were 8 *μ*g/mL (2 strains), 16 *μ*g/mL (4 strains), and 32 *μ*g/mL (2 strains).

*Salmonella *Typhimurium resistance to colistin was described by Sun et al. [3], who assessed spontaneous mutations in *Pmr*A and *Pmr*B genes in *S*. Typhimurium LT2 that present reduced susceptibility to colistin. They report that the mutation rate to colistin resistance was 0.6 × 10^6^ per cell generation, which was considered several times higher than mutations rates to other antibiotics, such as streptomycin, rifampicin, and nalidixic acid. The MIC values observed in these mutants (2.5 *μ*g/mL to 4 *μ*g/mL) increased 20 to 30 times comparing to susceptible strain (0,125 *μ*g/mL). Reports of colistin resistance frequency in wild *S. enterica* strains of animal origin and reports of resistance detection in other serotypes different from Typhimurium were not found in the literature. 

The MIC values identified in *S. enterica* resistant strains (4 *μ*g/mL and 8 *μ*g/mL) were lower than those observed in *E. coli*, but are still above the considered breakpoint and are 32 to 64 times higher than MIC observed in *S.* Typhimurium ATCC 14024 (0,125 *μ*g/mL). In this study colistin resistance was detected in wild *Salmonella* strains from serotype Typhimurium, London, Anatum, Bredeney and *S. enterica* subsp. *enterica* (O:4,5:-:1,2), suggesting that the large use of colistin in swine herds from Brazil is selecting resistant strains independent of serotype. Part of these *Salmonella* resistant strains was isolated from carcasses, lymph nodes and feces of pigs at slaughterhouses.

 Humans may obtain antimicrobial resistant bacteriaor resistance genes of animal origin directly via contact with animals, food of animal origin, or the environment. These bacteria may subsequently colonize humans or may transfer resistance genes to other bacteria during passage through the intestinal tract. The contribution of the animal reservoir to the burden of antimicrobial resistance in humans has not been quantified; however, the use of antimicrobial agents regarded as critically or highly important for use in humans should be avoided or minimized in food animals [16].

Considering the high use of colistin in animal production and the importance of this antimicrobial for the control of multiresistant Gramnegative nosocomial infections in humans, more intensive studies must be conducted to monitor the resistance in animal isolates and resistance mechanisms involved.

## Figures and Tables

**Table 1:** Distribution of MIC values of swine *E. coli* and *S. enterica* strains through agar dilution test against colistin.

Agar dilution	Number of strains with colistin MIC values (*μ*g/mL)											
	≤0.125	0.25	0.5	1	2	4	8	16	32	64	128	256
*E. coli*	23	51	40	4	0	[0	2	4	2	0	0	0]
*S. enterica*	0	1	59	30	8	[13	13	0	0	0	0	0]

The values inside [] represent resistant strains.

**Table 2:** Distribution of inhibition zone diameters swine *E. coli* and *S. enterica* strains through disk diffusion test against colistin.

Disk difusion	Number of strains with colistin inhibition zone values (mm)											
	8	9	10	11	12	13	14	15	16	17	18	19
*E. coli*	[0	0	4	0]	34	3	74	2	8	0	1	0
*S. enterica*	[0	0	5	0]	17	12	69	4	12	0	5	0

The values inside [] represent resistant strains.

**Table 3:** Distribution of colistin resistant strains among different *Salmonella enterica* serotypes isolated from swine.

Serotype	Strains	Number of resistant strains	
	No (%)	Agar dilution test	Disk diffusion test
*S.* Typhimurium	81 (65.3)	12	2
*S.* London	13 (10.5)	6	0
*S.* Anatum	11 (8.9)	3	0
*S. enterica* subsp. *enterica* (O:4,5:-:1,2)	8 (6.5)	4	3
*S*. Choleraesuis	7 (5.6)	0	0
*S*. Infantis	3 (2.4)	0	0
*S*. Bredeney	1 (0.8)	1	0

Total	124	26	5
